# Communications between Mitochondria, the Nucleus, Vacuoles, Peroxisomes, the Endoplasmic Reticulum, the Plasma Membrane, Lipid Droplets, and the Cytosol during Yeast Chronological Aging

**DOI:** 10.3389/fgene.2016.00177

**Published:** 2016-09-27

**Authors:** Pamela Dakik, Vladimir I. Titorenko

**Affiliations:** Department of Biology, Faculty of Arts and Science, Concordia UniversityMontreal, PQ, Canada

**Keywords:** yeast, chronological aging, interorganelle communications, macromolecular damage, cellular proteostasis, hormesis, programmed cell death, signal transduction

## Abstract

Studies employing the budding yeast *Saccharomyces cerevisiae* as a model organism have provided deep insights into molecular mechanisms of cellular and organismal aging in multicellular eukaryotes and have demonstrated that the main features of biological aging are evolutionarily conserved. Aging in *S. cerevisiae* is studied by measuring replicative or chronological lifespan. Yeast replicative aging is likely to model aging of mitotically competent human cell types, while yeast chronological aging is believed to mimic aging of post-mitotic human cell types. Emergent evidence implies that various organelle-organelle and organelle-cytosol communications play essential roles in chronological aging of *S. cerevisiae*. The molecular mechanisms underlying the vital roles of intercompartmental communications in yeast chronological aging have begun to emerge. The scope of this review is to critically analyze recent progress in understanding such mechanisms. Our analysis suggests a model for how temporally and spatially coordinated movements of certain metabolites between various cellular compartments impact yeast chronological aging. In our model, diverse changes in these key metabolites are restricted to critical longevity-defining periods of chronological lifespan. In each of these periods, a limited set of proteins responds to such changes of the metabolites by altering the rate and efficiency of a certain cellular process essential for longevity regulation. Spatiotemporal dynamics of alterations in these longevity-defining cellular processes orchestrates the development and maintenance of a pro- or anti-aging cellular pattern.

## Introduction

Studies of the budding yeast *Saccharomyces cerevisiae* have been instrumental in discovering genes, signaling pathways, and chemical compounds that influence cellular and organismal aging in evolutionarily distant eukaryotes (Fontana et al., 2010; Kaeberlein, 2010; Longo et al., 2012; Arlia-Ciommo et al., 2014a,b). These studies have revealed that the key aspects of the aging process and mechanisms of its modulation by certain genetic, dietary, and pharmacological interventions have been conserved in the course of evolution (Eisenberg et al., 2009; Fontana et al., 2010; Arlia-Ciommo et al., 2014a; Denoth Lippuner et al., 2014; Medkour et al., 2016a). One paradigm of aging in yeast is replicative aging. It is believed to imitate aging of mitotic human cell types capable of dividing (Steinkraus et al., 2008; Kaeberlein, 2010; Longo et al., 2012; Denoth Lippuner et al., 2014; McCormick et al., 2015), although recent findings suggest that yeast replicative aging may also serve as a suitable model for the aging of post-mitotic tissues and for the aging of whole organism in the nematode *C. elegans* and humans (Ghavidel et al., 2015; McCormick et al., 2015; Janssens and Veenhoff, 2016). A body of evidence supports the notion that diverse interorganelle communications influence yeast replicative aging (Beach et al., 2012; Hughes and Gottschling, 2012; Henderson et al., 2014; Janssens et al., 2015; Hughes et al., 2016). This evidence has been comprehensively discussed elsewhere (Jazwinski, 2012, 2013, 2014, 2015; Jazwinski and Kriete, 2012). Another paradigm of aging in yeast is chronological aging. It is likely to mimic aging of post-mitotic human cell types incapable of dividing (Burtner et al., 2009, 2011; Kaeberlein, 2010; Longo et al., 2012; Arlia-Ciommo et al., 2014a), although there is evidence that yeast chronological aging may converge with yeast replicative aging into a single aging process (reviewed in Arlia-Ciommo et al., 2014b; see also Mirisola and Longo, 2012; Murakami et al., 2012; Polymenis and Kennedy, 2012; Delaney et al., 2013; Molon et al., 2015). Recent findings indicate that many organelle-organelle and organelle-cytosol communications impact yeast chronological aging (Goldberg et al., 2009a; Titorenko and Terlecky, 2011; Beach et al., 2012; Beach and Titorenko, 2013; Leonov and Titorenko, 2013). Mechanisms underlying the essential roles of such intercompartmental communications in yeast chronological aging have begun to emerge. Here, we critically analyze recent progress in understanding these mechanisms.

## Spatiotemporal Dynamics of Intercompartmental Communications Define the Chronology of Cellular Aging in Yeast

**FIGURE 1:**
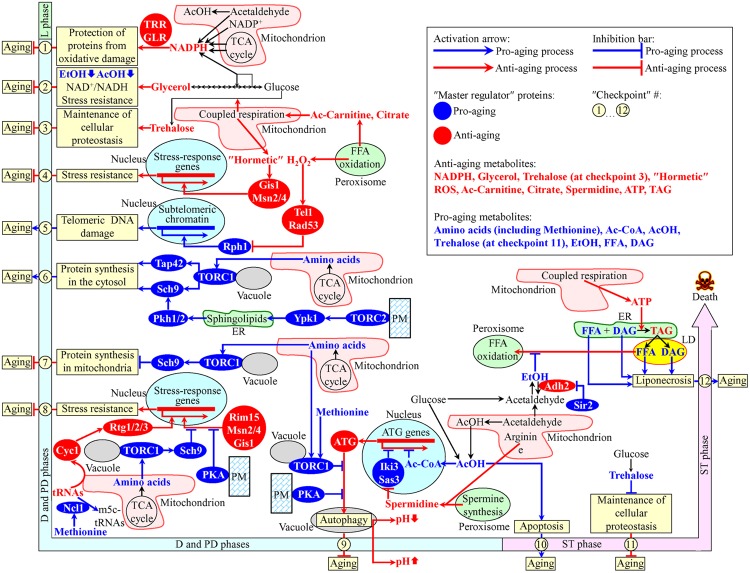
**A model for how various organelle-organelle and organelle-cytosol communications impact yeast chronological aging.** These communications involve movements of certain metabolites between various cellular compartments. Different changes in these metabolites are temporally restricted to longevity-defining periods called checkpoints. At each checkpoint, the changes of these metabolites are detected by certain master regulator proteins. Because each of the master regulator proteins modulates certain longevity-defining cellular processes, a coordinated in space and time action of these proteins orchestrates the development and maintenance of a pro- or anti-aging cellular pattern. See text for details. Ac-Carnitine, acetyl-carnitine; Ac-CoA, acetyl-CoA; AcOH, acetic acid; DAG, diacylglycerol; EtOH, ethanol; FFA, free (non-esterified) fatty acids; m5c-tRNAs, 5-methylcytosine tRNAs; PKA, protein kinase A; PM, the plasma membrane; ROS, reactive oxygen species; TAG, triacylglycerols; TCA, tricarboxylic; TORC, target of rapamycin complex; tRNAs, transfer RNAs.

Recent studies have revealed that various intercompartmental communications (i.e., organelle-organelle and organelle-cytosol) play essential roles in chronological aging of yeast cultured in media with glucose as the only carbon source (Beach and Titorenko, 2011; Beach et al., 2012, 2015a; Leonov and Titorenko, 2013; Medkour and Titorenko, 2016b). A model for how such communications impact yeast chronological aging is depicted schematically in **Figure 1**. Our model includes the notion that the longevity-defining intercompartmental communications involve unidirectional and bidirectional movements of a distinct set of metabolites between mitochondria and the cytosol, mitochondria and peroxisomes, mitochondria and the nucleus, peroxisomes and the nucleus, mitochondria and vacuoles, the endoplasmic reticulum (ER) and the plasma membrane (PM), the ER and the cytosol, the PM and the cytosol, the PM and vacuoles, the ER and lipid droplets (LD), and LD and peroxisomes (**Figure 1**). The intracellular concentrations of such metabolites and/or the rates of their movement between cellular compartments undergo age-related changes. In our model, different changes of the key metabolites are temporally restricted to several longevity-defining periods; the term “checkpoints” has been coined to describe these critical periods in yeast chronological lifespan (Burstein et al., 2012; Kyryakov et al., 2012; Arlia-Ciommo et al., 2014a; Beach et al., 2015a,b) (**Figure 1**). Most of these checkpoints occur early in life of chronologically aging yeast cells, during diauxic (D), and post-diauxic (PD) growth phases. Some of the checkpoints are late-life checkpoints that exists in the non-proliferative stationary (ST) phase of culturing. At each of these checkpoints, the changes of the key metabolites are detected by a distinct set of checkpoint-specific proteins called “master regulators” (Arlia-Ciommo et al., 2014a; Beach et al., 2015a). Our model further posits that each of these master regulators can respond to a change of the detected key metabolite by altering the rate and efficiency of a certain cellular process essential for longevity regulation (**Figure 1**). By establishing the rates and efficiencies of different longevity-defining cellular processes throughout chronological lifespan, the checkpoint-specific master regulators set up a pro- or anti-aging cellular pattern (Arlia-Ciommo et al., 2014a; Beach et al., 2015a).

At checkpoint 1, which exists early in D growth phase, two oxidative reactions of the pentose phosphate pathway in the cytosol and four enzymatic reactions in mitochondria create NADPH (Cai and Tu, 2012; Barral, 2013; Brandes et al., 2013; Arlia-Ciommo et al., 2014a) (**Figure 1**). NADPH provides reducing equivalents for the synthesis of amino acids, fatty acids, and sterols (Fraenkel, 2011; Cai and Tu, 2012). NADPH is also a donor of electrons for thioredoxin and glutathione reductase systems. Both these reductase systems contribute to the establishment and maintenance of an anti-aging cellular pattern because they protect many thiol-containing proteins from oxidative damage; such thiol-containing proteins reside in the nucleus, mitochondria and cytosol (Barral, 2013; Brandes et al., 2013) (**Figure 1**).

Glycerol is produced by glucose fermentation in the cytosol (Fraenkel, 2011). At checkpoint 2, glycerol plays an important role in the establishment and maintenance of an anti-aging cellular pattern by affecting the following cellular processes: (1) glucose fermentation to glycerol weakens its fermentation to ethanol and acetic acid, both known to be pro-aging metabolites in yeast; (2) glucose fermentation to glycerol enables to sustain the NAD^+^/NADH ratio that slows yeast chronological aging; and (3) glycerol increases resistance to acute oxidative, thermal, and osmotic stresses that accelerate yeast chronological aging (Burtner et al., 2009; Wei et al., 2009; Arlia-Ciommo et al., 2014a) (**Figure 1**).

In the cytosol of chronologically “young” yeast progressing through D and PD growth phases, the non-reducing disaccharide trehalose is synthesized from glucose (Goldberg et al., 2009b; Kyryakov et al., 2012). The rate of such synthesis sustains cellular trehalose homeostasis and is modulated by the efficiency of coupled mitochondrial respiration (Ocampo et al., 2012). The efficiency of such respiration is, in turn, modulated by the rate of peroxisome-to-mitochondria transfer of citrate and acetyl-carnitine (Epstein et al., 2001; Traven et al., 2001; Hiltunen et al., 2003; Titorenko and Terlecky, 2011; Arlia-Ciommo et al., 2014a). At checkpoint 3, trehalose is essential for maintaining an anti-aging pattern of cellular proteostasis because it attenuates the misfolding, aggregation and oxidative damage of newly synthesized polypeptides (Goldberg et al., 2009b; Kyryakov et al., 2012; Arlia-Ciommo et al., 2014a) (**Figure 1**).

During D and PD growth phases, the intracellular concentration of hydrogen peroxide (H_2_O_2_) in chronologically aging yeast depends on the efficiencies with which this major reactive oxygen species (ROS) is produced by and released from mitochondria and peroxisomes (Goldberg et al., 2010; Mirisola and Longo, 2013; Schroeder et al., 2013; Schroeder and Shadel, 2014). If the concentration of H_2_O_2_ at checkpoint 4 is sustained at a sub-lethal (“hormetic”) level, it elicits the establishment of an anti-aging cellular pattern by stimulating the master regulators Gis1, Msn2, and Msn4. In the nucleus, these three transcriptional factors activate expression of genes that encode proteins involved in heat-shock and DNA-damage responses, ROS decomposition, cell cycle progression and transition to quiescence, autophagy, maintenance of cell wall integrity, trehalose synthesis and degradation, glycogen synthesis and degradation, glycolysis and gluconeogenesis, the pentose phosphate pathway, glycerol and amino acid synthesis, ergosterol synthesis, maintenance of glutathione and thioredoxin homeostasis, methylglyoxal detoxification, maintenance of heavy metal ion homeostasis, potassium transport, and mitochondrial electron transport; these proteins are needed for resistance to thermal, oxidative, osmotic, low pH, carbon source starvation, sorbic acid, high ethanol concentration, and DNA-damage stresses (Martínez-Pastor et al., 1996; Schmitt and McEntee, 1996; Boy-Marcotte et al., 1998; Causton et al., 2001; Fabrizio et al., 2001) (**Figure 1**).

At checkpoint 5, H_2_O_2_ produced by and released from mitochondria and peroxisomes modulates a signaling pathway which includes the DNA damage response kinases Tel1 and Rad53 (both of which are anti-aging master regulators) and the histone demethylase Rph1 (a pro-aging master regulator) (Mirisola and Longo, 2013). If the concentration of H_2_O_2_ at this checkpoint is sustained at a hormetic level, it stimulates the Tel1-depenent phosphorylation/activation of Rad53, which in response phosphorylates and inactivates Rph1 (Schroeder et al., 2013; Schroeder and Shadel, 2014). The resulting inactivation of Rph1 establishes an anti-aging cellular pattern because it allows to attenuate the Rph1-dependent transcription of subtelomeric chromatin regions in the nucleus, thereby lessening the extent of telomeric DNA damage (Mirisola and Longo, 2013; Schroeder et al., 2013; Schroeder and Shadel, 2014) (**Figure 1**).

During D and PD growth phases, the amino acids aspartate, asparagine, glutamate, and glutamine are synthesized from intermediates of the TCA cycle in mitochondria (Fraenkel, 2011; Cai and Tu, 2012). After being released into the cytosol, these amino acids stimulate protein kinase (PK) activity of the TOR (target of rapamycin) complex 1 (TORC1) at the surface of vacuoles (Crespo et al., 2002; Powers et al., 2006; Jewell et al., 2013; Conrad et al., 2014; Shimobayashi and Hall, 2014; Swinnen et al., 2014). Following its activation, TORC1 acts as a pro-aging master regulator at checkpoint 6 by phosphorylating the nutrient-sensory PK Sch9 and the Tap42 protein. Once phosphorylated, Sch9 and Tap42 accelerate the pro-aging process of protein synthesis in the cytosol by stimulating ribosome biogenesis and augmenting translation initiation (Hinnebusch, 2005; Huber et al., 2009; Conrad et al., 2014; Swinnen et al., 2014; Eltschinger and Loewith, 2016) (**Figure 1**). The TOR complex 2 (TORC2) at the PM also functions as a pro-aging master regulator at checkpoint 6. If activated, TORC2 phosphorylates the PK Ypk1. After being phosphorylated, Ypk1 stimulates the synthesis of complex sphingolipids in the ER. These sphingolipids then stimulate the PKs Pkh1 and Pkh2, both of which in response phosphorylate Sch9 to intensify the pro-aging process of protein synthesis in the cytosol (Roelants et al., 2004; Liu et al., 2005; Urban et al., 2007; Huang et al., 2014; Eltschinger and Loewith, 2016; Teixeira and Costa, 2016) (**Figure 1**).

At checkpoint 7, the amino acids aspartate, asparagine, glutamate, and glutamine are released from mitochondria and activate TORC1 at the surface of vacuoles. Active TORC1 sets off a pro-aging cellular pattern by phosphorylating Sch9, which then attenuates the anti-aging process of protein synthesis in mitochondria (Bonawitz et al., 2007; Pan and Shadel, 2009; Conrad et al., 2014; Shimobayashi and Hall, 2014; Swinnen et al., 2014) (**Figure 1**).

At checkpoint 8, the efflux of the amino acids aspartate, asparagine, glutamate and glutamine from mitochondria, resulting activation of TORC1 at the vacuolar surface and subsequent phosphorylation of Sch9 cause a retention of the nutrient-sensory PK Rim15 in the cytosol (Wanke et al., 2008; Smets et al., 2010). Because under such conditions Rim15 cannot enter the nucleus, it is unable to stimulate Msn2, Msn4, and Gis1; these three transcriptional activators can orchestrate an anti-aging transcriptional program in the nucleus only if they are stimulated by Rim15 (Wanke et al., 2008; Smets et al., 2010; Conrad et al., 2014; Shimobayashi and Hall, 2014; Swinnen et al., 2014) (**Figure 1**). Furthermore, protein kinase A (PKA) activity at the cytosolic leaflet of the PM also contributes to the establishment of a pro-aging cellular pattern at checkpoint 8. This PK activity inhibits nuclear import of Msn2 and Msn4, thus turning off an anti-aging transcriptional program driven – in a Rim15-dependent manner – by these two transcriptional activators (Medvedik et al., 2007; Lee et al., 2008; Smets et al., 2010; Conrad et al., 2014) (**Figure 1**). Moreover, a study on a methionine restriction-induced delay of yeast chronological aging implies that the excess of methionine can elicit a pro-aging cellular pattern at checkpoint 8 by activating the tRNA methyltransferase Ncl1 in the cytosol (Johnson and Johnson, 2014). This decreases the concentration of non-methylated tRNAs, attenuates the efflux of cytochrome *C* (Cyc1) from mitochondria and mitigates nuclear import of the cytosolic Rtg1/Rtg2/Rtg3 heterotrimeric transcriptional factor, which is required for the stimulation of an anti-aging transcriptional program in the nucleus (Johnson and Johnson, 2014) (**Figure 1**).

If TORC1 at the surface of vacuoles is activated by the release of the amino acids aspartate, asparagine, glutamate, and glutamine from mitochondria at checkpoint 9, active TORC1 phosphorylates the autophagy-initiating protein Atg13 (Laplante and Sabatini, 2012; Conrad et al., 2014; Shimobayashi and Hall, 2014; Swinnen et al., 2014). At this checkpoint, Atg13 can also be phosphorylated by PKA kinase activity confined to the cytosolic face of the PM (Yorimitsu et al., 2007; Stephan et al., 2009, 2010). The TORC1- and PKA-driven phosphorylation of Atg13 at checkpoint 9 inhibits autophagosome formation in the cytosol, thus suppressing the anti-aging process of autophagy (Yorimitsu et al., 2007; Stephan et al., 2009, 2010; Shimobayashi and Hall, 2014) (**Figure 1**). Furthermore, a study on the methionine restriction-induced delay of yeast chronological aging revealed that the excess of methionine in the cytosol can trigger a pro-aging cellular pattern at checkpoint 9 because it weakens autophagy, either by stimulating TORC1 at the vacuolar surface or by attenuating autophagosome formation in the cytosol (Ruckenstuhl et al., 2014). Such methionine-driven weakening of autophagy accelerates aging by decreasing the extent of vacuolar acidification and by increasing acetic acid accumulation in cultural medium (Wu et al., 2013; Johnson and Johnson, 2014) (**Figure 1**). Moreover, mitochondria, peroxisomes and the cytosol house individual reactions for the synthesis of the polyamine spermidine (Minois et al., 2011; Beach and Titorenko, 2013; Minois, 2014). At checkpoint 9, spermidine inhibits the histone acetyltransferases Iki3 and Sas3 (Eisenberg et al., 2009). Although such spermidine-driven inhibition of Iki3 and Sas3 causes global decline in the acetylation of histone H3 and silencing of numerous genes in the nucleus, histones in the promoter regions of several *ATG* (autophagy) genes get acetylated under these conditions (Eisenberg et al., 2009; Morselli et al., 2009, 2011; Madeo et al., 2010). The resulting selective activation of transcription of these genes at checkpoint 9 promotes the anti-aging process of autophagy (**Figure 1**). Also, a fraction of acetic acid in the cytosol can be imported into the nucleus and then converted into acetyl-CoA in the Acs2-dependent reaction (Eisenberg et al., 2014). At checkpoint 9, this acetyl-CoA selectively represses transcription of nuclear *ATG* genes, thus suppressing the anti-aging process of autophagy (Eisenberg et al., 2014; Schroeder et al., 2014) (**Figure 1**).

Chronologically aging yeast cells produce acetic acid as follows: (1) it is generated as the alternative product of glucose fermentation in the cytosol; and (2) it is formed in the Ald4-dependent reaction in mitochondria, from which acetic acid can be released into the cytosol (Burtner et al., 2009; Fraenkel, 2011; Longo et al., 2012; Arlia-Ciommo et al., 2014a). At the late-life checkpoint 10 in ST phase, a pool of acetic acid in the cytosol accelerates yeast chronological aging because it elicits an age-related form of apoptotic programmed death (Burtner et al., 2009, 2011; Longo et al., 2012; Murakami et al., 2012; Arlia-Ciommo et al., 2014a) (**Figure 1**).

While at the early-life checkpoint 3 trehalose is essential for maintaining an anti-aging pattern of cellular proteostasis (see above), this non-reducing disaccharide sets off a pro-aging cellular pattern at the late-life checkpoint 11 in ST phase. This is because in chronologically “old” yeast cells, which do not grow or divide, trehalose covers hydrophobic amino acid side chains of misfolded and unfolded proteins (Goldberg et al., 2009b; Kyryakov et al., 2012; Arlia-Ciommo et al., 2014a). Such side chains are needed to be recognized by a group of molecular chaperones that help to refold these misfolded and unfolded proteins (Chen et al., 2011; Lindquist and Kelly, 2011; Taylor and Dillin, 2011; Kim et al., 2013). By competing with molecular chaperones for binding to such clusters of hydrophobic amino acids, trehalose attenuates the anti-aging process of maintaining cellular proteostasis (Goldberg et al., 2009b; Kyryakov et al., 2012; Arlia-Ciommo et al., 2014a) (**Figure 1**).

At the late-life checkpoint 12 in ST phase, the excessive accumulation of free (non-esterified) fatty acids (FFA) and diacylglycerol (DAG) in cellular membranes accelerates yeast chronological aging because it triggers an age-related form of programmed cell death called liponecrosis (Goldberg et al., 2009a,b; Arlia-Ciommo et al., 2014a, 2016; Richard et al., 2014) (**Figure 1**). ATP, which is produced mainly in mitochondria, slows age-related liponecrosis by providing energy needed for the detoxification of FFA in the ER through the incorporation of FFA into triacylglycerols (TAG) and other neutral lipids (Arlia-Ciommo et al., 2014a; Richard et al., 2014; Sheibani et al., 2014). Ethanol, a product of glucose fermentation, accelerates age-related liponecrosis by suppressing peroxisomal oxidation of FFA that are generated in LD due to lipolysis of TAG and other neutral lipids (Goldberg et al., 2009a,b; Arlia-Ciommo et al., 2014a; Beach et al., 2015a). The sirtuin deacetylase Sir2 promotes ethanol accumulation by inactivating the Adh2 isoform of alcohol dehydrogenase, which is required for ethanol catabolism (Fabrizio et al., 2005) (**Figure 1**).

## Conclusion

In this review, we analyzed mechanisms through which temporally and spatially coordinated organelle-organelle and organelle-cytosol communications impact yeast chronological aging. Our analysis indicates that these communications are integrated into a convoluted network involving unidirectional and bidirectional movements of certain metabolites between cellular compartments. Different changes in the intracellular concentrations and the rates of movement of these metabolites are restricted to critical longevity-defining periods of chronological lifespan called checkpoints. Certain proteins known as master regulators can detect the changes of the key metabolites at each of these checkpoints. The checkpoint-specific master regulator proteins contribute to setting up a pro- or anti-aging cellular pattern because each of these proteins modulates certain longevity-defining cellular processes. Future work will aim at understanding how certain dietary and pharmacological interventions known to delay aging can modulate information flow within the intricate network of intercompartmental communications.

## Author Contributions

PD and VT wrote the text. VT prepared the figure.

## Conflict of Interest Statement

The authors declare that the research was conducted in the absence of any commercial or financial relationships that could be construed as a potential conflict of interest.
